# Real-world evidence of a novel tetravalent immunoglobulin Y effectiveness and safety in patients with the refractory Helicobacter pylori infection

**DOI:** 10.1186/s12879-024-09498-4

**Published:** 2024-06-27

**Authors:** Nan Hao, Bo Liu, Meng Zhao, Mingming Lu, Feiyi Chen, Jialu Kang, Xiaojun Tang, Yong Zhang, Chengxue Dang

**Affiliations:** 1 Department of Surgical Oncology, The First Affiliated Hospital of Xi’an Jiaotong University, Xi’an, Shaanxi China; 2 Department of Hepatobiliary Surgery, The First Affiliated Hospital of Xi’an Jiaotong University, Xi’an, Shaanxi China; 3 Key Laboratory of Antibody Technique, Nanjing Medical University, Nanjing, Jiangsu China

**Keywords:** Helicobacter pylori, Refractory, Tetravalent IgY, Eradication, Real-world

## Abstract

**Background:**

Refractory Helicobacter pylori (*H*. pylori) infection inevitably increase the difficulty of drug selection. Here, we described our experience with the use of a novel tetravalent IgY against *H*. pylori for the treatment of patients with refractory *H.* pylori infection.

**Methods:**

Patients were randomly assigned to receive the standard quadruple therapy (amoxicillin, clarithromycin, omeprazole and bismuth potassium citrate ) for 2 weeks or 250 mg of avian polyclonal IgY orally twice a day for 4 weeks. The binding efficacy of IgY to *H*. pylori antigens was detected by western blotting_13_. C-urea breath test was performed to evaluate the eradication therap’s efficacy. The side effects of IgY were evaluated via various routine tests. The questionnaire was used to gather clinical symptoms and adverse reactions.

**Results:**

Western blot analysis showed that tetravalent IgY simultaneously bind to VacA, HpaA, CagA and UreB of *H*. pylori. Tetravalent IgY had an eradication rate of 50.74% in patients with refractory *H*. pylori and an inhibition rate of 50.04% against DOB (delta over baseline) of _13_C-urea. The symptom relief rate was 61.76% in thirty-four patients with clinical symptoms, and no adverse reactions were observed during tetravalent IgY treatment period.

**Conclusions:**

Polyclonal avian tetravalent IgY reduced *H*. pylori infection, and showed good efficacy and safety in the treatment of refractory *H.* pylori infection patients, which represented an effective therapeutic option of choice for patients with refractory *H.* pylori infection.

**Supplementary Information:**

The online version contains supplementary material available at 10.1186/s12879-024-09498-4.

## Introduction

The infection rate of Helicobacter pylori (*H*. pylori) has exceeded 50% worldwide, and even reached 80% in developing countries [1]. *H*. pylori infection is a common cause of gastritis and gastric ulcers, and a risk factor for gastric cancer [2]. China alone accounts for half of the approximate 1 million new cases of gastric carcinoma globally each year, and almost all cases are considered to be related to *H.* pylori infection [3, 4]. Gastric cancer seriously affect the survival quality of patients and cause a tremendous economic burden, due to its high mortality rate [5]. Therefore, eradication of *H*. pylori is essential to prevent and treat gastritis, gastric ulcer, gastric cancer and so on.

With relevant in-depth researches, the guidelines and consensus on the eradication of *H*. pylori have been constantly updated. Currently, the accepted treatment regimen is the standard quadruple therapy (two kinds of antibiotics including metronidazole, amoxicillin, clarithromycin, tetracycline or levofloxacin combined with proton pump inhibitors and bismuth agents), and the course of treatment has changed from 7 days to 10–14 days [6–10]. Although the standard quadruple therapy achieves a high *H.* pylori eradication rate, refractory *H.* pylori patients who failed in previous treatment twice or more still accounts for more than 10% in developing countries [11, 12]. Extensive reviews reported antibiotic resistance was the main factor for initial treatment failure in patients with refractory *H.* pylori infection (13–14). Recently, the drug resistance of *H*. pylori is growing, due to the abuse of antibiotics in China. Studies have shown that the resistance of *H*. pylori to metronidazole has reached 81% [15], and to levofloxacin and clarithromycin has reached 18% and 44%, respectively [16]. With the increasing resistance of *H*. pylori to antibiotics, partial *H*. pylori-positive patients lacked an effective treatment [17, 18]. Meanwhile, the increasing resistance also complicates the re-eradication of patients with *H*. pylori recurrence. In addition, repeated treatment will increase the economic burden of patients and cause a tremendous waste of medical resources [19]. Besides, patients may miss medicine since the time or frequency of administration of each medication in standard quadruple is disaffinity and the medication cycle lasts for 2 weeks, which might interfere the eradication of *H.* pylori. So it’s necessary to establish a simplified approach to eradicate *H.* pylori without considering the antibiotic resistance and obtain higher compliance without omission or discontinuation of medicine.

Egg yolk immunoglobulin Y (IgY) is a kind of polyclonal antibody with molecular weight of 180 kDa. Its preparation is convenient, and it has the characteristics of acid resistance and heat resistance and is not easy to occure drug resistance. Previous studies have shown that egg yolk IgY can be taken orally for passive immunization and plays an important role in the prevention and treatment of gastrointestinal bacterial, virus and parasite infections [20–24], suggesting that the use of egg yolk IgY specific to *H*. pylori may have potential advantages in *H*. pylori eradication. Shin JH et al. [20] confirmed that egg yolk IgY against *H*. pylori whole-cell lysates inhibited the growth of *H*. pylori and reduced the accumulation of inflammatory cells in the stomach of Mongolian gerbils infected with *H.* pylori. Later, studies by Suzuki H et al. [25] showed that the [13]C-urea breath test value of *H*. pylori-positive volunteers who took anti-*H*. pylori urease IgY for four weeks was significantly lower than that before treatment, and these volunteers had no significant adverse effects during the treatment period. In addition, Borhani K et al. [26] studied the inhibitory effect of anti- *H*. pylori neutrophil-activating protein (NAP) IgY on *H*. pylori attachment to gastric adenocarcinoma cell lines, and found that anti-*H*. pylori NAP IgY could significantly inhibit the attachment of *H*. pylori to AGS cells. All the above studies suggest that egg yolk IgY might be used to prevent and treat *H*. pylori infection, especially in refractory patients who have developed antibiotic resistance.

However, the egg yolk IgY against *H*. pylori whole-cell lysates may cross-react with other bacteria including intestinal flora while inhibiting *H*. pylori, thereby reducing its effectiveness and specificity [27]. In addition, the amount of monovalent egg yolk IgY against *H*. pylori that is used in the treatment may lead to an increase of treatment cost. Nevertheless, using multivalent egg yolk IgY may solve these problems. Given that vacuolating cytotoxin A (VacA) [28], *H*. pylori adhesin A (HpaA) [29], cytotoxin-associated gene A (CagA) [30] and Urease B (UreB) [30] are major toxins produced and secreted by *H*. pylori, which contribute to colonization and virulence of *H*. pylori in multiple ways. Thereby, in our study, we reported the real-world effectiveness and safety of polyclonal avian tetravalent IgY against *H*. pylori antigens (including VacA, HpaA, CagA and UreB) in the treatment of patients with refractory *H*. pylori infection.

## Methods

### Binding efficacy of IgY

Egg yolk IgY (obtained from Unik Biology Medicine Co, Ltd.) was isolated and purified from hens immunized with *H*. pylori antigens (VacA, HpaA, CagA and UreB). The purified VacA, HpaA, CagA and UreB antigens (2 mg/ml) were separated with SDS-PAGE. Protein marker (Thermo Fisher, 26,616) was used to as a reference for protein molecular weight. After electrophoresis, the gel was attached to nitrocellulose membrane. After 20 V semi-dry transfer for 1 h, the nitrocellulose membrane was removed and washed with PBST for 3 times. Then the membrane was blocked with 50 g/L BSA solution at 37 °C for 1.5 h and washed with PBST. 10 µg/ml polyclonal avian tetravalent IgY solution was added and incubated at 37 °C for 2 h. Goat anti-chiken IgY (Thermo Fisher, 35,552, 1:2500) was added and incubated at 37 °C for 1 h. The membrane was washed with PBST and stained with DAB solution for 5–10 min in the dark. Finally, the membrane was washed with distilled water and the staining was stopped. IMAGE LAB 5.1 software was used to deal the images.

### Patients and IgY therapy

We performed a prospective observational study describing the use of polyclonal avian tetravalent IgY or the standard quadruple in patients with refractory *H.* pylori infection in the First Affiliated Hospital of Xi ‘an Jiaotong University. Ethics approval was granted by The Review Boards of Shaanxi Province Clinical Cancer Research Center affiliated to the First Affiliated Hospital of Xi’an Jiaotong University (No. SX-ZLLC-2019-03). The study was performed in accordance with the ethical standards as laid down in the 1964 Declaration of Helsinki and its later amendments or comparable ethical standards. Patients provided written informed consent before enrolment.

**Fig. 1:**
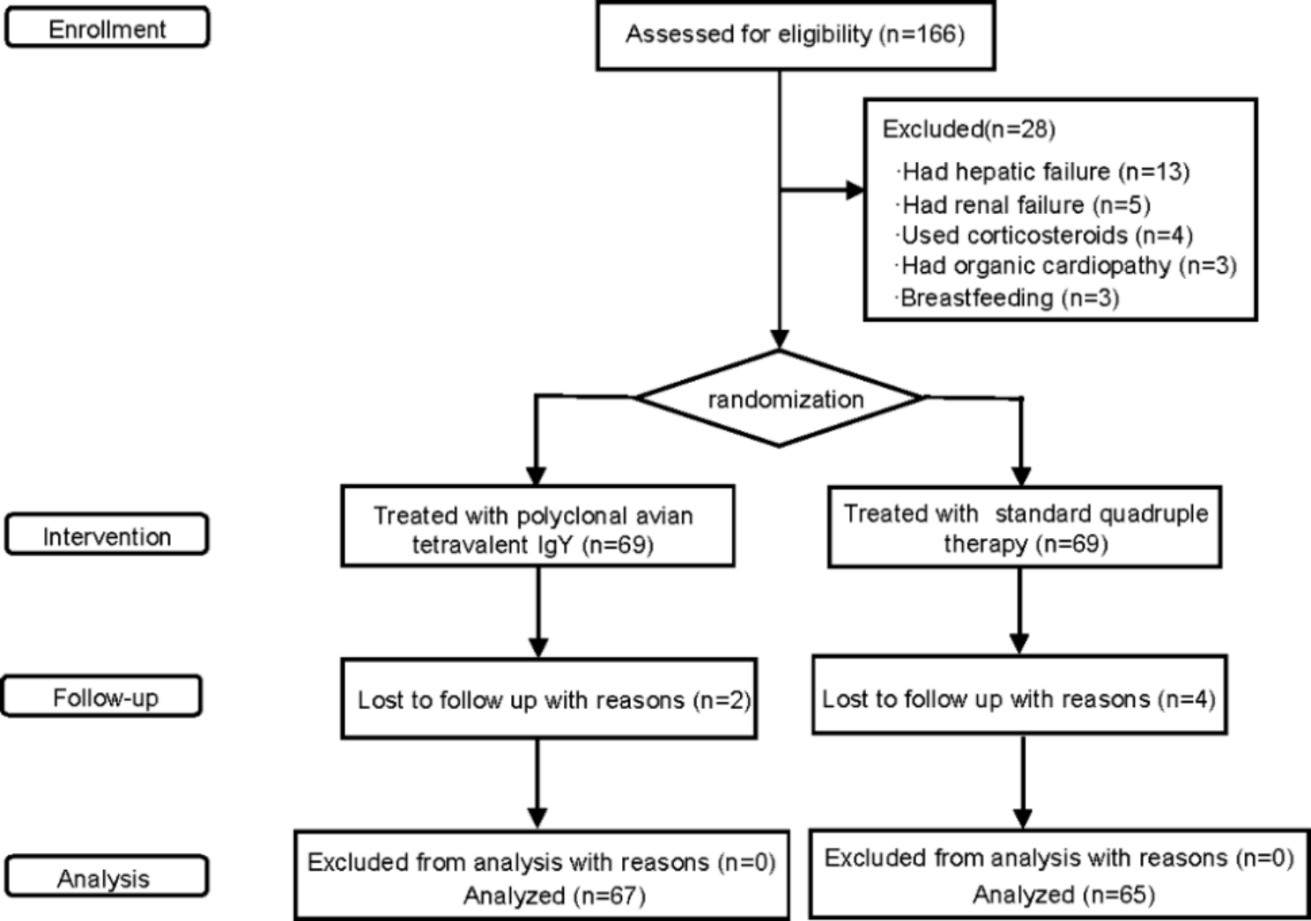
Flow chart of patients enrollment, exclusion, randomization, intervention, follow-up and analysis

Patients were eligible if they were 18 to 80 years of age and if they failed in previous therapy twice or more and and it has been 1 month since the last treatment ended. One hundred and sixty-six patients with refractory *H.* pylori infection between May 2021 and April 2022 were eligible in this study. Patients were excluded if they had any of the following criteria: active bacterial (except *H.* pylori) infection or mycosis with systemic effects; pregnant or breastfeeding; hepatic failure or renal failure; systemic administration of corticosteroids; unstable angina or myocardial infarction within 6 months before enrolment. Twenty-eight patients were excluded because they did not conform the criteria and six were lost to follow-up. Finally, one hundred and thirty-two patients with refractory *H.*pylori infection between May 2021 and April 2022 were included in this study (Fig. 1).

Sixty-seven patients were treated orally with egg yolk IgY (250 mg, one capsule) against *H.* pylori 30 min before lunch twice daily for 4 weeks and sixty-five patients were treated orally with standard quadruple therapy (amoxicillin (500 mg, tow capsules) clarithromycin (500 mg, one tablet), omeprazole (20 mg, one capsule) and bismuth potassium citrate (110 mg, two capsules)) 30 min before lunch and supper for 2 weeks. For [13]C-urea urea breath test, each patient drank 2.1 g of citric acid, 150 mg of aspartame and 125 mg of [13]C-urea in 100 ml of water. A premixed urea solution was composed and then a baseline breath test was performed, and the cut-off value for positive versus negative was a delta-over-baseline (DOB) of 4.0. The following baseline characteristics of patients were recorded: age, gender, manifestations, the value of DOB of [13]C-urea breath test and the results of blood routine, stool routine, urine routine, hepatic function and renal function examinations. Adverse reaction monitoring included allergy, abdominal pain, diarrhea, constipation, nausea, vomiting, headache, palpitations and so on.

### Outcomes

^13^C-urea urea breath test was performed to evaluate the eradication therapy’s efficacy at the day before the start of treatment and 4 weeks after the end. Blood routine, stool routine, urine routine, hepatic function and renal function examinations were performed during treatment and within 4 weeks after therapy. The improvement of clinical symptoms and the incidence of adverse events were measured during treatment and within 4 weeks after therapy.

### Statistical analysis

Data analysis was performed by GraphPad Prism 8.3.0 (GraphPad Software, Inc.). Descriptive data are expressed as mean ± standard deviation. Student t-test was used for the comparison between two groups. *P*-value < 0.05 was considered to be statistically different. P-value < 0.01 was considered to be significant statistically different. P-value < 0.001 was considered to be very significant statistically different.

## Results

### Binding of egg yolk IgY to *H*. Pylori antigen

**Fig. 2:**
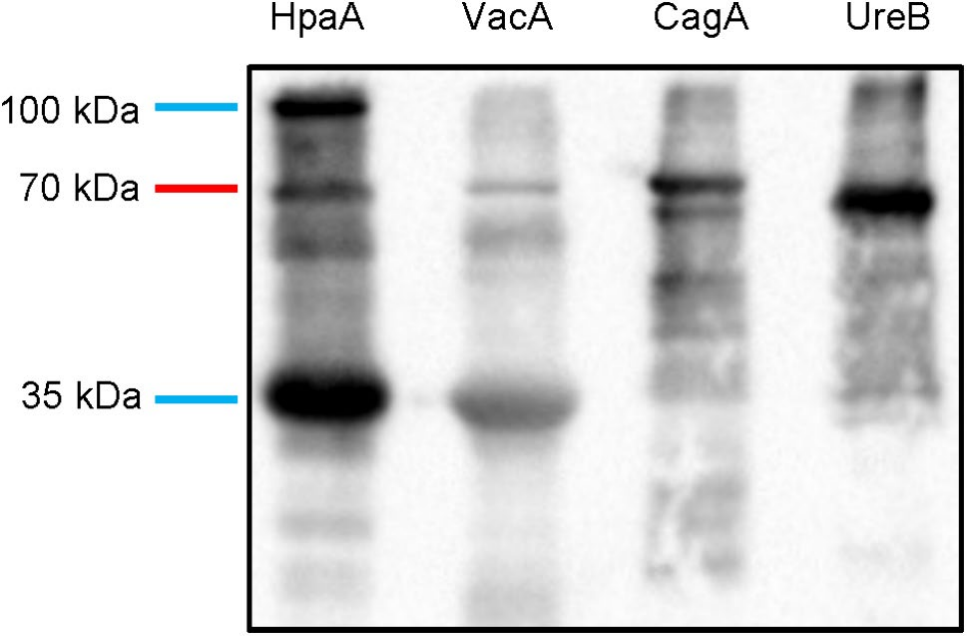
Western blot analysis showed that polyclonal avian tetravalent IgY could be specific to *H*. pylori antigens. *H.* pylori: Helicobacter pylor

We performed western blot analysis to detect whether egg yolk IgY against *H*. pylori could simultaneously recognize and react with the four antigens of *H*. pylori. The results showed that several dominant bands with reactivity to egg yolk IgY were observed against *H*. pylori antigens (Fig. 2). The corresponding bands appeared at the Mr = 80 kDa, 70 kDa, 35 kDa and 33 kDa respectively (Fig. 2), indicating that polyclonal avian tetravalent IgY could simultaneously recognize and react with CagA, UreB, HpaA and VacA of *H*. pylori. These results indicated that egg yolk IgY against was specific against *H*. pylori.

### Patient characteristics

A total of 138 people were eligible and 6 of them were lost to follow-up, the other conditions were as follows: 65 people completed the standard quadruple therapy, 67 people completed the polyclonal avian tetravalent IgY treatment. The baseline characteristics of patients are shown in Table 1. Thirty-four (52.31%) patients were male and the median age of these patients was 56.0 (23.0–73.0) years old in standard quadruple therapy group. Thirty-five (52.24%) patients were male and the median age of these patients was 53.0 (25.0–72.0) years old in polyclonal avian tetravalent IgY therapy group. Of these, 67 patients showed different clinical manifestations, including abdominal pain, abdominal distension, acid regurgitation and so on Table 1.

**Table 1:** The baseline characteristics of patient with standard quadruple treatment

Variable	Standard quadruple	IgY	*P*-Value
Age	56(23–73)	53(25–72)	NS
Male, No (%)	34(52.31%)	35(52.24%)	NS
Clinical symptoms, No (%)			NS
Abdominal pain	9(13.85%)	7(10.45%)	NS
Abdominal distension	2(3.08%)	4(6.00%)	NS
Acid regurgitation	7(10.77%)	8(11.84%)	NS
Bitter taste	4(6.15%)	2(3.00%)	NS
Dyspepsia	1(1.54%)	3(4.48%)	NS
Eructation	2(3.08%)	3(4.48%)	NS
Esophageal foreign body sensation	3(4.62%)	1(1.50%)	NS
Loose stool	1(1.54%)	1(1.50%)	NS
Halitosis	1(1.54%)	3(4.48%)	NS
Insomnia	3(4.62%)	2(3.00%)	NS

### The efficacy and safety of egg yolk IgY in patient with *H*. Pylori recurrence

During the treatment period, the regimen was well tolerated, and no adverse reactions (allergy, abdominal pain, diarrhea, constipation, nausea, vomiting, headache, palpitations and so on) or complications were observed.

**Fig. 3:**
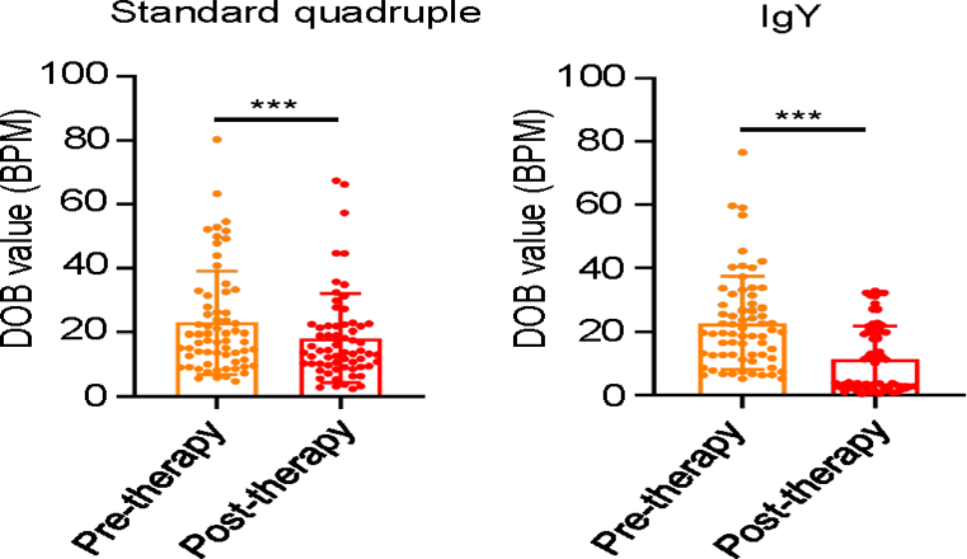
DOB value of patients with refractory *H.* pylori infection before and after treatment of standard quadruple therapy or polyclonal avian tetravalent IgY against *H.* pylori. *H.* pylori: Helicobacter pylori; DOB: delta over baseline. ***:*P*<0.001

**Fig. 4:**
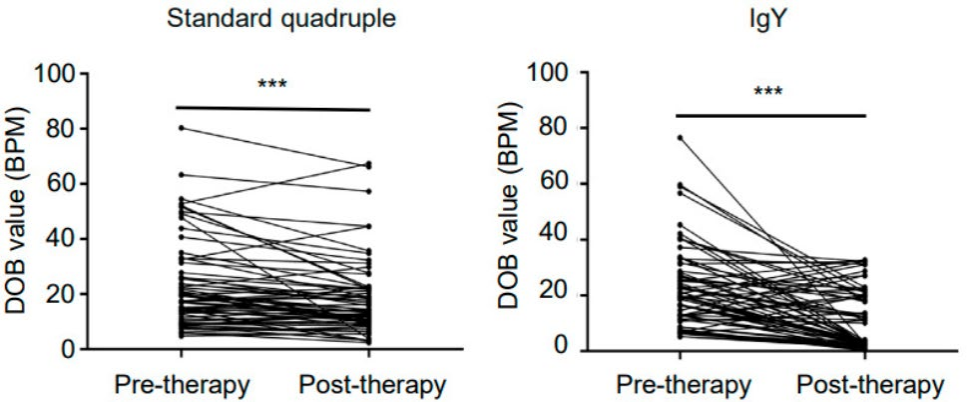
The trend of DOB values in patients with refractory *H.* pylori infection before and after treatment of standard quadruple therapy or polyclonal avian tetravalent IgY against *H.* pylori. *H.* pylori: Helicobacter pylori; DOB: delta over baseline. ***:*P*<0.001

We used [13]C-urea breath test to detect the efficiency of the standard quadruple theragy or egg yolk IgY against *H*. pylori. In general, both the standard quadruple theragy and egg yolk IgY reduced the DOB of [13]C-urea breath test in patients (Fig. 3).While reduction rate of DOB in egg yolk IgY group reached 50.04%, much higher than that of standard quadruple theragy group (20.85%) (Table 2). As Fig. 4 showed, the DOB of [13]C-urea breath test in patients demonstrated a decreasing trend, and the trend of egg yolk IgY group was more obvious than standard quadruple theragy group. Meanwhile, The eradication rate of *H.* pylori in egg yolk IgY group was 50.74%, while that in standard quadruple theragy group was only 7.7% (Table 3), indicating that for refractory *H.* pylori infection patients, egg yolk IgY has a better therapeutic effect than the standard quadruple therapy. Besides, after oral administration of egg yolk IgY against H. pylori for 4 weeks, 21 (61.76%) patients achieved clinical remission, which was approximate twice as high as that of the standard quadruple therapy group (30.30%) (Table 4).

**Table 2:** Reduction rate of DOB in patients with standard quadruple treatment or IgY treatment

Treatment		Reduction rate (%)
standard quadruple		20.85%
IgY		50.04%

Abbreviation: *H*.pylori: Helicobacter pylori

**Table 3:** Eradication rate of *H*. pylori in patients with standard quadruple treatment or IgY treatment

Treatment	Number of patients	Eradication rate (%)
standard quadruple	65	7.70% (05/65)
IgY	67	50.74% (34/67)

*Abbreviation H*.pylori: Helicobacter pylori

**Table 4:** Relief rate of symptoms in patients with standard quadruple treatment or IgY treatment

Treatment	Number of patients	Relief rate (%)
standard quadruple	33	30.30% (10/33)
IgY	34	61.76% (21/34)

*Abbreviation *H.pylori: Helicobacter pylori

Taken together, polyclonal avian tetravalent IgY (against *H*. pylori) was very effective in suppression of *H*. pylori infection, and no side effects were noted during the treatment period.

## Discussion

Due to a series of problems such as antibiotic resistance and poor patient compliance, the prevention and treatment of *H*. pylori has become complicated. Therefore, it is necessary to find new strategies to eradicate *H*. pylori. Previous studies have shown that egg yolk IgY could effectively inhibit *H*. pylori [20, 23–25, 27]. Besides, IgY generally does not induce allergic reactions because it neither binds to rheumatism factor, protein A/G, and Fc receptors on cell surface, nor activates the complement system [31, 32]. In addition, egg yolk IgY is not easily digested by trypsin and chymotrypsin [33]. Therefore, to investigate the role of egg yolk IgY in eradication of *H*. pylori will be meaningful. In the present study, we confirmed the efficacy and safety of polyclonal tetravalent IgY against *H*. pylori antigens (VacA, HpaA, CagA and UreB) patients with *H*. pylori recurrence, in order to provide a new alternative strategy for *H*. pylori eradication.

Oral administration egg yolk IgY can provide passive immunity for patients by inhibiting bacteria in several ways: (1) inhibiting bacterial enzyme activity; (2) neutralizing toxins produced by bacteria; (3) blocking bacterial attachment to cells. Previous studies have shown that egg yolk IgY inhibits the activity of *H*. pylori in *vitro* and in *vivo* [23, 34]. In 2002, Shin JH et al. firstly reported that egg yolk IgY obtained from hens immunized with *H*. pylori could significantly reduce the degree of lymphocyte and neutrophil infiltration in the stomach of Mongolian gerbils infected with *H*. pylori [20]. But then, they confirmed that IgY obtained from hens immunized with *H*. pylori whole-cell lysates could not only react with *H*. pylori but also with other intestinal flora, thereby reducing the efficacy of IgY against *H*. pylori [27]. Subsequently, to address this deficiency, researchers prepared egg yolk IgY specific to a single pathogen of *H*. pylori and made breakthroughs. In 2004, Shin JH et al. further confirmed that anti-urease activity of IgY specific to *H*. pylori urease produced using the synthetic peptide was very similar to that of IgY against *H*. pylori whole-cell lysates [35]. Meanwhile, clinical trials in humans had shown that oral administration of egg yolk IgY against *H*. pylori urease significantly inhibited *H*. pylori infection [21, 25]. In addition to anti-*H*. pylori urease IgY, researchers prepared egg yolk IgY specific to NAP [26], VacA [24], Sydney Strain 1 (SS1) and recombinant UreI-UreB protein (rIB) [36], and found that these specific IgY targeting *H*. pylori antigens had a strong inhibitory effect on *H*. pylori and could significantly reduce *H*. pylori infection.

However, the problem is that a large amount of IgY specific to a single *H*. pylori antigen leads to increased treatment costs. In addition, previous studies had shown that anti-*H*. pylori vaccines targeting a single subunit could not prevent the variant immune escape of antigenic *H*. pylori, while multivalent vaccines could enhance the immune intensity and breadth, thereby enhancing the antibacterial efficiency of anti-*H*. pylori vaccines [37]. Similarly, IgY targeting multiple *H*. pylori antigens may be more effective in eradication of *H*. pylori infection. Given that VacA, HpaA, CagA and UreB are major virulence factor of *H*. pylori, we prepared polyclonal avian tetravalent IgY targeting these four antigens. In our study, western blot results showed that multivalent egg yolk IgY simultaneously recognized and reacted with VacA, HpaA, CagA and UreB, indicating that it could inhibit *H*. pylori by binding these four antigens.

We further conducted a prospective observational study of oral egg yolk IgY or standard quadruple therapy in patients with refractory *H.* pylori infection to determine its role in eradication of *H*. pylori. We found that the DOB values of patients in egg yolk IgY group were significantly lower than that in standard quadruple therapy group, suggesting that egg yolk IgY did inhibit *H*. pylori and excel standard quadruple therapy. Among these, 34 (50.74%) patients showed negative [13]C-urea breath test results (DOB value less than 4.0), indicating that IgY has the potential to eradicate *H.* pylori. In addition, 61.76% of 34 patients with clinical symptoms were significantly relieved after treatment. Of note, during the treatment period, no patient had any adverse reactions. All patients showed well tolerance to IgY treatment, indicating that IgY had good safety and might be used for clinical eradication of *H*. pylori.

In addition, egg yolk IgY could avoid the side effects caused by the standard quadruple therapy, and might be more suitable for the eradication of *H.*pylori in special patients, such as pregnant women, children and the elderly. Since antibiotics might cause damage to the fetal growth and development [38], *H.* pylori-positive pregnant women who cannot receive standard quadruple therapy suffered additional distress. Egg yolk IgY cannot pass through the gastric barrier, so it will not affect fetus. Therefore, the application of IgY might be a good alternative strategy for the treatment of *H*. pylori-positive pregnant women. Children were exposed to a high *H.* pylori infection status, and further damage of infection remains unclear [39]. And administration of antibiotics could interfered with the maturation and destabilization of intestinal flora in children, leading to several diseases [40, 41]. In our study, no side effects were observed in treatment with egg yolk IgY during the treatment period. Therefore, IgY might be used for *H.*pylori-positive children with gastrointestinal symptoms. Further clinical trials should be conducted to confirmed the efficacy and safety of polyclonal avian tetravalent IgY in children and pregnant women, which we will do soon.

## Conclusion

In this study, our results confirmed that polyclonal avian tetravalent IgY could simultaneously combine with *H*. pylori and reduce *H*. pylori infection, and showed good efficacy and safety in the treatment of patients with refractory *H.* pylori infection.

### Electronic supplementary material

Below is the link to the electronic supplementary material.

Supplementary Material 1

Supplementary Material 2

## Data Availability

No datasets were generated or analysed during the current study.

## Abbreviations

H. pylori: Helicobacter pylori
DOB: Delta over baseline
