# Supplementary figures and images for: Real-world evidence of a novel tetravalent immunoglobulin Y effectiveness and safety in patients with the refractory Helicobacter pylori infection
Source: BMC Infect Dis. 2024 Jun 27;24:647. doi: 10.1186/s12879-024-09498-4 (PMC11210110; doi:10.1186/s12879-024-09498-4)

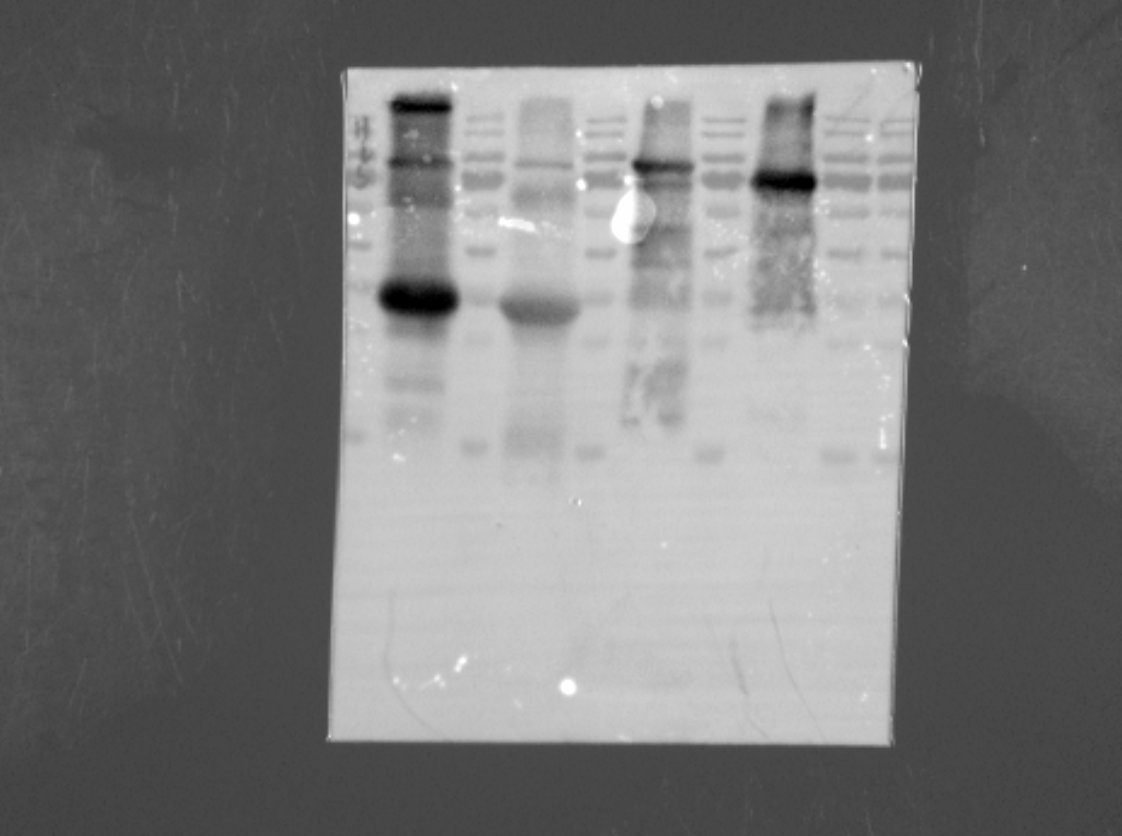

Supplement: Supplementary file 1 — Supplementary Material 1 [file 12879_2024_9498_MOESM1_ESM.tif]

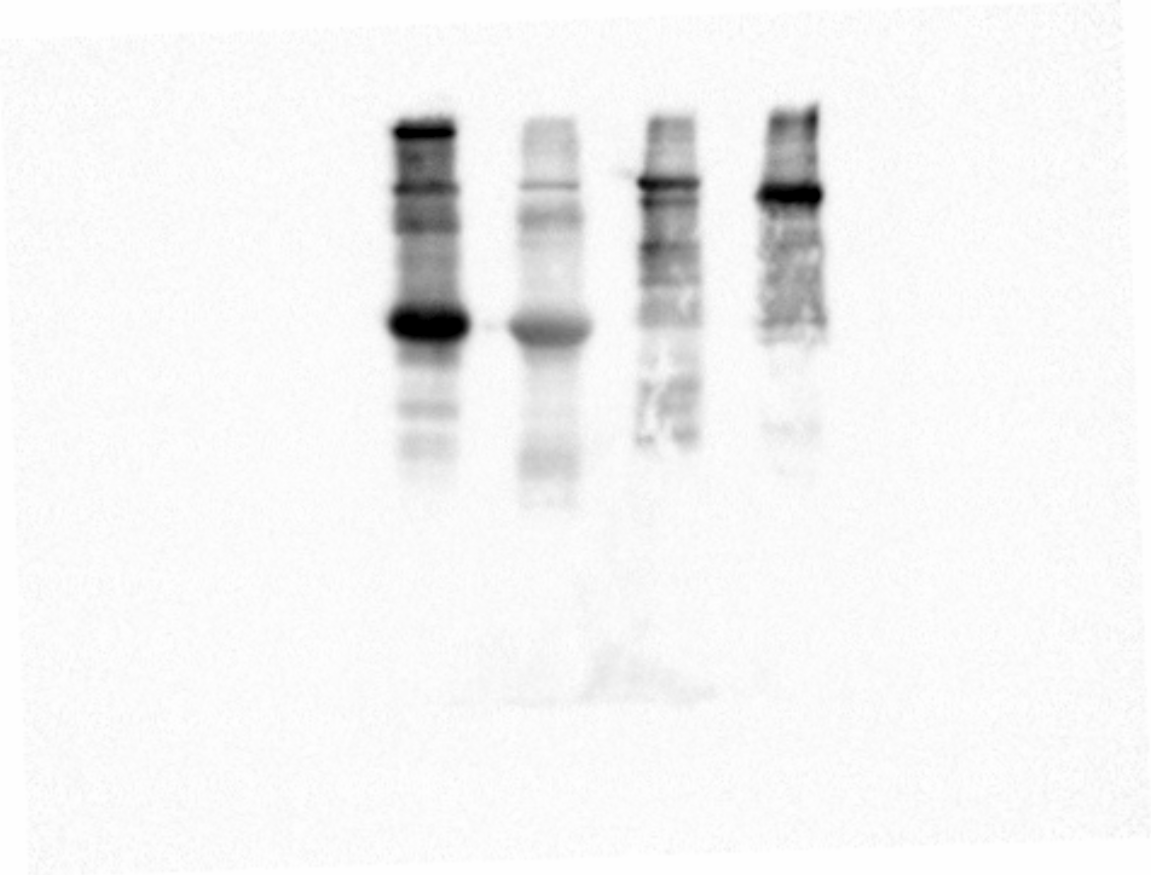

Supplement: Supplementary file 2 — Supplementary Material 2 [file 12879_2024_9498_MOESM2_ESM.tif]
